# A novel method for the collection of highly developmental murine immature oocytes

**DOI:** 10.1016/j.mex.2020.100879

**Published:** 2020-04-19

**Authors:** Ahmed M. Taiyeb

**Affiliations:** a Division of Physiology and Pharmacology, School of Medicine, University of Kurdistan-Hewler, Erbil 44001, Kurdistan, Iraq; b Barz IVF Center for Embryo Research and Infertility Treatment, Koyah Street, Brayati, Erbil 44001, Kurdistan, Iraq

**Keywords:** Cilostazol, Gavage, Germinal vesicle, Metaphase I, Superovulation

## Abstract

Isolation of germinal vesicle (GV) or metaphase I (MI) oocytes from large antral follicles, using a 30 gauge needle, in mice is a common method for the retrieval of immature oocytes from ovaries. However, this method depends entirely on the experience and judgment of the investigator. It is possible that not all of the isolated immature oocytes are from large antral follicles nor necessarily represent the cohort of oocytes that would be perfectly developed and consequently ovulated upon hormonal stimulation. Here, we administered an FDA approved phosphodiesterase 3A inhibitor, named cilostazol, in superovulated mice to result in the ovulation of GV or MI oocytes, depending on time and frequency of administration. The presented method results in mice ovulating GV or MI oocytes, which can be recovered from the oviduct without the investigator's judgment mentioned above. This method does not only result in immature oocytes with high yield, health, synchronized maturation, and competence levels but also is time and labor efficient. It also permits for physiological selections of a cohort of immature oocytes that would be entirely developed and eventually ovulated, as opposed to the conventional method.

• Complete superovulation
• Administration of cilostazol at different times
• Recovery of ovulated immature oocytes from oviducts

## Graphical abstract

**Figure:**
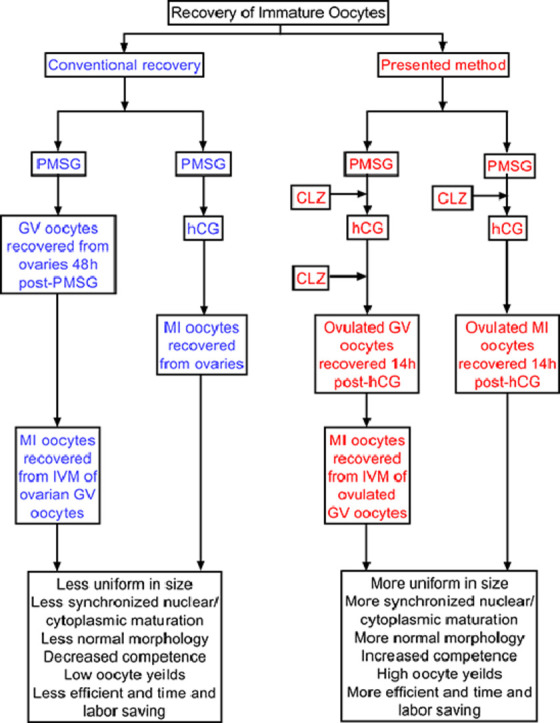

**Table:** Specifications Table

Subject Area	Biochemistry, Genetics and Molecular Biology
More specific subject area	*Reproductive biology*
Method name	*Recovery of immature oocytes using cilostazol*
Name and reference of original method	*N/A*
Resource availability	*N/A*

## Background

Collection of germinal vesicle (GV) oocytes from large antral follicles in hyperstimulated mice require experiences to select antral follicles under a microscope before puncturing the follicle and retrieving of GV oocytes. This is sometimes followed by the screening of oocytes based on morphology and diameter, and usually, mouse oocytes of less than 60µm are excluded, especially when the research is not designed to study outcomes following different oocyte sizes. Shortcomings with the current technique include oocyte yield and the possibility of collecting oocytes that are not from large antral follicles. Moreover, the observed antral follicles under a microscope do not always represent the oocytes that will be ovulated. This method also results in ovarian GV oocytes with different types of chromatin configurations, cortical granule and mitochondrial distribution, transcriptional activities, and sizes [5,10,17,20].

Metaphase I (MI) oocytes are usually obtained via *in vitro* maturation (IVM) of ovarian GV oocytes or collected directly from ovaries after the administration of human chorionic gonadotropin (hCG) in superovulated mice. The rate of MI oocytes obtained from IVM of ovarian GV oocytes depends on the type of culture medium, animal species, original size of GV oocyte or follicle used, and state of cumulus cells [3,4,9,11,13]. Retrieval of MI oocytes directly from ovaries has the same limitations mentioned with the collection of ovarian GV oocytes, in addition to the need to determine the time course required for germinal vesicle breakdown (GVBD). While some investigators have reported a range of 4-9h post-ovulatory stimulus for GVBD to occur, others have reported times shorter than 3h for GVBD to occur depending on the mouse strain [[2], [6], [7], [8]].

Oocytes, upon isolation from follicles, can undergo spontaneous meiotic maturation *in vitro* [12]. Meiotic maturation is believed to occur before cytoplasmic maturation. The lack of time synchronization between those two maturational components is believed to cause deficiencies in oocyte development and competence. For instance, oocytes collected in stimulated cycles are more readily fertilized after preincubation than are oocytes inseminated immediately after collection, indicating cytoplasmic maturation subsequent to the polar body extrusion [14]. Moreover, temporal arrest of spontaneous meiotic maturation of GV oocytes *in vitro* using phosphodiesterase 3 (PDE3) inhibitors is found to increase oocyte meiotic competence [1,17,18,21]. Previously, we showed that oral administration of cilostazol, a PDE3A inhibitor, in superovulated mice resulted in the ovulation of oocytes of different meiotic stages based on cilostazol dose, frequency of administration, and times of administration and collection of ovulated oocytes [19]. Administration of cilostazol before the hCG injection allowed superovulated mice to ovulate MI oocytes, whereas administration of cilostazol in divided doses around the time of hCG injection resulted in the ovulation of GV oocytes. We also found that when the collection of immature oocytes was delayed, the ovulated oocytes were able to resume meiotic maturation in the oviduct, indicating a reversible arrest of oocytes at the GV stage [19]. We also reported that the administration of cilostazol in superovulated mice resulted in synchronized cytoplasmic and nuclear maturation and improved competence [17,18]. The aim of this report is to detail this method of collection of immature oocytes in mice.

## Materials

Cilostazol

Dimethyl sulfoxide (DMSO)

Gavage feeding tube for mice

Insulin syringe

Pregnant mare serum gonadotropin (PMSG)

Human chorionic gonadotropin (hCG)

Oocyte recovery medium (e.g., human tubal fluid culture media supplemented with 4.5% (v/v) fetus bovine serum (FBS) and HEPES)

Stereomicroscope

## Procedure

For recovery of GV oocytes:

(1) Female Swiss Webster mice were primed intraperitoneally with 5 IU PMSG to stimulate the development of multiple follicles.
(2) Primed mice with PMSG were intraperitoneally injected with 5 IU of hCG, 47 h post-PMSG, to induce ovulation
(3) Superovulated mice with PMSG and hCG were then gavaged two times with 7.5mg cilostazol as follow:
  (a) The first 7.5mg cilostazol dose was dissolved in a warm 0.1mL DMSO and vortexed for one minute. The prepared dose is then aspirated in an insulin syringe and a gavage tube is placed to the syringe and gavaged to the animal at the same time of hCG injection. The amount of cilostazol left in the gavage tube should be determined in advance in order to adjust the final required volume of cilostazol to be delivered.
  (b) A second dose of cilostazol, prepared as mentioned above, is also administered to the animal 6 h post-hCG injection.
  (c) Alternatively, 7.5 mg cilostazol dissolved in 0.1mL DMSO can be administered 4h pre-hCG and 2h post-hCG in superovulated mice.
(4) Mice were sacrificed 13–14 h post-hCG using cervical dislocation after an appropriate anesthesia.
(5) Following the peritoneal incision, the ovaries, oviducts, and a part from the uterine horn are placed in a warm 2 mL HTF medium supplemented with HEPES, 4.5% FBS, and 4.2 μM cilostazol to block spontaneous oocyte meiotic maturation if required [16].
(6) The distended portion of the ampulla is then punctured using a 30 gauge needle under a stereomicroscope to release oocytes with enclosed cumulus cells at the GV stage.

For recovery of MI oocytes:

(1) Female Swiss Webster mice were primed intraperitoneally with 5 IU PMSG to stimulate the development of multiple follicles.
(2) Primed mice with PMSG were then intraperitoneally injected with 5 IU of hCG, 47 h post-PMSG, to induce ovulation.
(3) Superovulated mice with PMSG and hCG were then gavaged one time with 7.5mg cilostazol. Briefly, 7.5mg cilostazol dose dissolved in a warm 0.1mL DMSO is prepared as mentioned above and is administered to the animal (gavaged) at the same time of hCG injection or 4h pre-hCG.
(4) Mice were sacrificed 13-14 h post-hCG using cervical dislocation after an appropriate anesthesia.
(5) Following the peritoneal incision, the ovaries, oviducts, and a part from the uterine horn are placed in a warm 2 mL HTF medium supplemented with HEPES, 4.5% FBS, and 4.2 μM cilostazol to block spontaneous oocyte meiotic maturation if required.
(6) The distended portion of the ampulla is then punctured using a 30 gauge needle under a stereomicroscope to release oocytes with enclosed cumulus cells at the MI stage.
(7) Alternatively, MI oocytes can also be recovered from IVM of ovulated GV resultant from superovulated mice treated with the two doses of cilostazol as mentioned above.

## Method validation

Collection of immature oocytes from superovulated mice treated with cilostazol has resulted in immature oocytes that are more (a) uniform in size, (b) ready to undergo GVBD or first polar body emission, (c) synchronized with nuclear and cytoplasmic development, (d) competent, (e) and capable of producing live births than did control immature or mature oocytes [17], [18], [19]. The presented method is also characterized by ease of collection and processing, time-saving, and convenience in comparison to the conventional method. Finally, the cilostazol method results in high oocyte yield per mouse in comparison to immature oocytes recovered using the conventional method [15].

## Declaration of Competing Interests

The authors declare that they have no known competing financial interests or personal relationships that could have appeared to influence the work reported in this paper.
